# The complete chloroplast genome of *Epimedium davidii* Franch. (Berberidaceae)

**DOI:** 10.1080/23802359.2019.1704641

**Published:** 2020-01-08

**Authors:** Qianru Yang, Xiang Liu, Cheng Zhang, Yu Yao, Yanjiao Luo, Guoan Shen, Baolin Guo

**Affiliations:** a Institute of Medicinal Plant Development, Chinese Academy of Medical Science, Peking Union Medical College, Beijing, PR China;; b Chongqing Academy of Chinese Materia Medica, Chongqing, PR China;; c Shanxi Medical University, Taiyuan, China

**Keywords:** Chloroplast genome, *Epimedium davidii*, Berberidaceae

## Abstract

*Epimedium davidii*, which belongs to Berberidaceae, is mainly distributed in the southwest of China. In this study, the complete chloroplast genome of *E. davidii* was sequenced and assembled. The circular genome is 159,715 bp in length, which comprises a large single-copy region (LSC, 85,862 bp), a small single-copy region (SSC, 17,081 bp), and a pair of inverted repeat regions (IRa and IRb, 28,386 bp). The chloroplast genome of *E. davidii* contains 112 unique genes, of which 78 protein-coding genes, 30 tRNA genes, and 4 rRNA genes. Phylogenetic analysis indicated that *E. davidii* was closely related to *Epimedium acuminatum*.

The *Epimedium* L. plants in the Berberidaceae family have been used as traditional Chinese herbs for more than 2000 years. The leaves of *Epimedium*, Epimedium folium (EF), exhibit beneficial pharmacological effects including reinforcing the yang, improving immunological function, and treating cardiovascular diseases, etc. (Ma et al. 2011; Liu et al. 2019 Yang et al. 2019). Due to many controversies on the taxonomic and phylogenetic relationship of *Epimedium* family, more attention has been drawn to characterize the genetic diversity with modern molecular techniques (Li et al. 2015). DNA barcodes (*rbcL*, *psbA*-*trnH*, *ITS*, and *ITS2*) were used in identifying *Epimedium* species, but the classification and phylogeny of *Epimedium* family were still poorly solved (Guo et al. 2018). Besides, the application of chloroplast genome to identify species and determine phylogenetic relationship has been proven to be effective (Nguyen et al. 2015). Up to now, the complete chloroplast genome of five *Epimedium* species have been reported (Zhang et al. 2016; Liu et al. 2019), but more *Epimedium* species need to be further evaluated. In this study, we reported the complete chloroplast genome of *E. davidii* Franch. that is a unique species with some special floral appearance, aiming to provide more genomic information for the systematic and evolutionary studies.

In this study, *E. davidii* sample was collected from the Baoxing County of Sichuan province (China; 30°35′N, 102°52′E). A voucher specimen (18036) was deposited at the Herbarium of the Institute of Medicinal Plant (IMPLAD), Beijing, China. Total genomic DNA was extracted from the fresh leaves of *E. davidii* using the modified CTAB method (Doyle and Doyle 1987). The high-quality DNA was sheared to the size of 300 bp for the shotgun library construction. The sequencing was performed on an Illumina Novaseq PE150 platform (Illumina Inc., San Diego), and 150 bp paired-end reads were generated. The filtered reads were assembled into the complete chloroplast genome using the program GetOrganelle version1.5 (Jin et al. 2018) with *E. acuminatum* chloroplast genome (GenBank accession number: NC_029941) as a reference. The annotation of the chloroplast genome was conducted through the online program CPGAVAS 2 (Shi et al. 2019), followed by manual correction if required. The annotated genomic sequence has been registered in GenBank with an accession number (MN621353).

The chloroplast genome of *E. davidii* is 159,715 bp in length, which consists of a large single-copy region (LSC, 85,862 bp), a small single-copy region (SSC, 17,081 bp), and a pair of inverted repeat regions (IRa and IRb, 28,386 bp). The total GC content of *E. davidii* chloroplast genome is 38.81%, while the corresponding GC content of LSC, SSC, and IR regions is 37.34%, 32.79%, and 42.85%, respectively. The chloroplast genome of *E. davidii* contains 112 unique genes, including 78 protein-coding genes, 30 tRNA genes, and 4 rRNA genes. Intron-exon structure analysis indicated that nine protein-coding genes and five tRNA genes contained one intron, while three genes (*ycf3*, *clpP*, and *rps12*) had two introns. Eight protein-coding genes (i.e. *rpl2*, *rpl22*, *rpl23*, *rps7*, *rps12*, *rps19*, *ndhB*, and *ycf2*), seven tRNA genes (i.e. *trnI*-CAU, *trnL*-CAA, *trnV*-GAC, *trnI*-GAU, *trnA*-UGC, *trnR*-ACG, and *trnN*-GUU), and four rRNA genes (i.e. *rrn4.5S*, *rrn5S*, *rrn16S*, and *rrn23S*) are duplicated in the IR regions. Besides, one tRNA gene (*trnQ*-UUG) is duplicated in the LSC regions.

To identify the phylogenetic relationship of *E. davidii*, 17 complete chloroplast genomes of Berberidaceae species were used to reconstruct a maximum-likelihood (ML) phylogenetic tree using RAxML version 8.2.10 (Stamatakis 2014), with *Aconitum contortum* as the outgroup (Figure 1). Phylogenetic analysis indicated that *E. davidii* is closely related to *E*. *acuminatum*. The complete chloroplast genome of *E. davidii* provides useful perspectives into the evolutionary patterns in Berberidaceae family.

**Figure 1.**
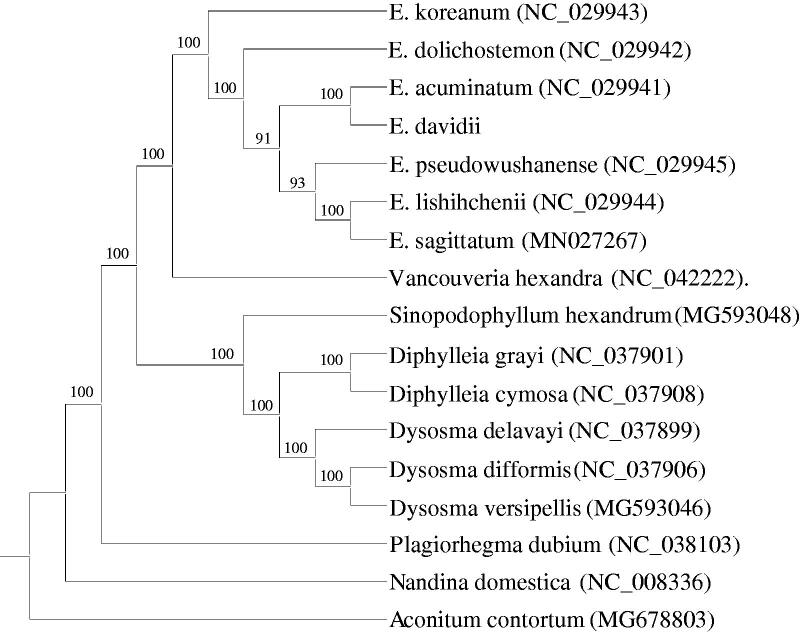
Phylogenetic tree reconstruction using maximum likelihood (ML) method based on the complete chloroplast genome of 17 species, with *Aconitum contortum* as the outgroup. Numbers above the lines represent ML bootstrap values.
